# Individual-Level Modeling of Depressive Symptom Severity Using Smartphone and Wearable Data: Time-Aware 1-Year Study With Feature-Group Contributions

**DOI:** 10.2196/99204

**Published:** 2026-08-25

**Authors:** Simon Schreynemackers, Johannes Leimhofer, Milica Petrovic, Hanna Reich, Sascha Ludwig, Andreas Dominik, Dominik Heider, Ulrich Hegerl, the MONDY Consortium, Mohamed Alhaskir

**Affiliations:** 1 German Foundation for Depression and Suicide Prevention, Leipzig, Germany; 2 Research Centre of the German Foundation for Depression and Suicide Prevention, Department of Psychiatry, Psychosomatic Medicine and Psychotherapy, University Hospital Frankfurt, Frankfurt, Hesse, Germany; 3 Institute of Computer Science, Faculty of Mathematics and Natural Sciences, Heinrich Heine University Düsseldorf, Universitätsstraße 1, Düsseldorf, North Rhine-Westphalia, 40225, Germany, +49 69 6301 86342; 4 Institute for Applied Informatics, Leipzig University, Leipzig, Saxony, Germany; 5 Department of Mathematics Natural Sciences and Informatics, Life Science Informatics Group, University of Applied Sciences, Giessen, Germany; 6 Institute of Medical Informatics, University of Münster, Münster, North Rhine-Westphalia, Germany; 7 Goethe Research Professorship, Department for Psychiatry, Psychosomatics and Psychotherapy, University Hospital Frankfurt, Frankfurt, Hesse, Germany; 8 See Acknowledgments

**Keywords:** major depressive disorder, digital phenotyping, smartphone data, wearable devices, idiographic modeling, feature importance, mobile phone

## Abstract

**Background:**

Smartphones and wearables can continuously capture behavioral and physiological data in everyday life. Such mobile-sensing data may help track depressive symptoms more closely than occasional retrospective questionnaires, but prior findings have been mixed. Inconsistent findings do not preclude the presence of predictive relationships in specific individuals or time periods. In addition, it remains unclear which broader sensor domains, rather than single features, contribute most to prediction at the individual level.

**Objective:**

The objective of this study is to evaluate whether smartphone and wearable data improve prospective prediction of daily depressive symptom severity beyond a person-specific baseline and, where improvement is observed, to identify contributing sensor feature groups.

**Methods:**

Data from the MONDY (secure and open platform for AI-based health care apps) n-of-1 study were analyzed, including adults with recurrent major depressive disorder who provided up to 12 months of daily self-reported depressive symptom ratings, together with continuous smartphone and smartwatch data. To capture individual-specific patterns, separate participant-specific (idiographic) linear and nonlinear models were developed for each individual. Performance was evaluated using repeated temporally separated training and test periods against a person-specific baseline model predicting the mean symptom score from the training period. Explainability analyses were restricted to models showing improvement over baseline, and summarized at the level of sensor feature groups.

**Results:**

Among 11 participants with sufficient data, linear models improved predictive performance over the person-specific baseline in 8 (73%) participants, and nonlinear models did so in a different set of 8 (73%) participants. Improvements over the person-specific baseline, expressed as reductions in mean absolute error (change in mean absolute error relative to baseline), ranged from 0.05 to 1.23 points for linear models and from 0.12 to 1.07 points for nonlinear models on the adapted Patient Health Questionnaire-2 scale. Predictive performance varied substantially between individuals, and although nonlinear models exhibited greater aggregate feature-group contributions, this did not translate into a consistent overall predictive advantage over linear models. Feature-group importance profiles were heterogeneous, and no single sensor domain dominated across all participants.

**Conclusions:**

Multimodal smartphone and wearable data can provide individual-specific predictive signals for daily depressive symptom severity in a subset of patients when evaluated under prospective, time-aware conditions. Both predictive performance and contributing sensor domains vary markedly across individuals, and no single modeling approach is uniformly superior. These findings suggest that previously reported associations in mobile-sensing research, often arising from heterogeneous study designs and feature representations, may not consistently translate into prospective prediction, and highlight the importance of evaluation frameworks that assess predictive signal at the temporal individual-participant level.

## Introduction

### Background

Major depressive disorder is a highly heterogeneous and recurrent condition in which symptom burden can fluctuate substantially within the same person over time [1]. In clinical practice, severity is commonly tracked with self-report questionnaires administered at comparatively sparse intervals (eg, weekly to monthly) to guide measurement-based care [2]. However, retrospective symptom reports are susceptible to recall biases and cognitive heuristics, and they often capture different information than high-frequency, momentary assessments [3]. These limitations have motivated interest in objective, ecologically valid markers that can be collected continuously in daily life.

Mobile sensing (MS) aims to infer mental states from passively captured behavioral and physiological signals such as activity [4-8], sleep-wake patterns [4,7-10], social interaction proxies [4], heart rate variability (HRV) [7,9], and speech characteristics [8,11] recorded by smartphones and wearables. Recent reviews conclude that multimodal passive sensing can, in principle, relate to depressive symptoms and trajectories, but results vary widely across studies due to differences in data quality, sensor coverage, preprocessing choices, outcome definitions, and evaluation procedures [12-14]. Long-term MS data are often missing for behavioral rather than purely technical reasons: missingness not at random may reflect withdrawal or reduced engagement, and disengagement from digital monitoring has been associated with symptom severity or relapse risk [15,16]. Such adherence-related signal is often not treated as an explicit domain in model evaluation and interpretation. Importantly, evidence increasingly suggests that personalized (idiographic) modeling and multimodal integration can improve predictive performance compared with one-size-fits-all approaches, consistent with the person-specific nature of depression dynamics [17]. However, this evidence spans a wide range of outcome definitions (eg, daily symptom severity [4], symptom profiles [6], binary or categorical states [5,9,11,18], or longer-horizon symptom change [10]) and model classes (from linear and mixed models [4,8] to nonlinear machine learning and deep learning [5-7,9-11,18]), which complicates direct comparisons and may contribute to inconsistent findings [12,17].

Even when idiographic models outperform one-size-fits-all baselines on average, prospective performance can remain unstable because the sensor-symptom relationship is not fixed over time [12,19]. One key methodological reason is that the association between sensors and symptoms is plausibly time-varying: routines, treatment effects, seasonality, and life events can change the behavioral meaning of the same sensor feature over time. This phenomenon is closely related to concept drift and model degradation in nonstationary environments [19]. Complementing this, intensive longitudinal clinical research shows that within-person symptom dynamics and relationships among clinically relevant variables can shift over time rather than remaining stable [20]. Together, these considerations imply that a single static model can appear worse than the mean under nonstationarity in prospective evaluation even when informative structure exists intermittently or only in specific phases, making time-aware validation and rolling assessment critical for determining whether there is reliable predictive signal.

Beyond predictive performance, interpretability represents a second major challenge. Many mobile-sensing studies report importance at the level of individual predictors (eg, coefficient lists [4,8], impurity-based rankings [10,21], and SHAP [Shapley Additive Explanations] summaries [9,18]), but high-dimensional sensing pipelines routinely produce large sets of correlated features. In such settings, per-feature attribution can be unstable and difficult to communicate, especially if explanations rely on assumptions that are strained by correlated predictors. For example, widely used SHAP variants can yield misleading attributions when feature dependence is not handled appropriately [22]. Relatedly, some work derives patient-specific physiological biosignatures from wearables, but these approaches typically summarize selected predictors rather than quantifying domain contributions via prospective, performance-based perturbations over time [7].

A pragmatic and clinically interpretable alternative is to shift from single-feature explanations toward sensor-domain (feature-group) explanations, where predictors are aggregated into interpretable modality groups (eg, activity regularity, HRV, sleep, communication, app interaction, and audio), because domains align with clinically meaningful behaviors (sleep, activity, and social withdrawal). Grouped importance supports cross-model comparability because it can be implemented in a model-agnostic manner and summarized consistently across linear and nonlinear approaches. Methodologically, permutation-based grouped importance has been formalized and reviewed as a general framework for model-agnostic explanation at the feature-group level [23]. This perspective is particularly relevant for the multimodal estimation of depressive symptom severity using smartphones and wearable data, where the scientific question often concerns which domains are informative rather than which single derived feature dominates [8].

Against this background, it remains necessary to examine whether multimodal passive sensing can predict day-to-day symptom variation under conditions that more closely reflect real-world use. In particular, even when idiographic models outperform one-size-fits-all approaches on average, prospective performance may remain unstable because sensor-symptom relationships are likely to vary over time. At the same time, a key limitation of the existing literature is that predictive performance is often evaluated using nonprospective or insufficiently time-aware validation strategies and only rarely benchmarked against simple person-specific baseline models. As a result, it remains unclear whether reported associations translate into meaningful predictive signal beyond trivial reference models under real-world longitudinal conditions.

To address these limitations, this study combines (1) strictly prospective, time-aware evaluation, (2) explicit comparison against a person-specific baseline, and (3) model-agnostic, domain-level explainability restricted to cases with demonstrated predictive value using rolling, block-wise permutation-based attribution that respects temporal structure. This design enables a more rigorous assessment of when and for whom MS provides incremental predictive information, while linking interpretability analyses explicitly to demonstrated predictive signal.

### Objective

This study aims to evaluate whether multimodal passive sensing features can predict day-to-day variation in depressive symptom severity at the individual level using up to 12 months of daily self-reported symptoms and continuous smartphone and smartwatch data from adults with recurrent major depressive disorder participating in the MONDY (secure and open platform for AI-based health care apps) trial [24]. To address this aim, elastic net (EN) regression as the primary linear modeling approach and random forest (RF) regression as a complementary nonlinear approach were applied to examine whether passive sensing features provide predictive information beyond a simple person-specific baseline and whether nonlinear modeling improves detection of such participant-specific signal.

The following research questions are addressed:

Primary research question 1 (RQ1): In how many patients do passive sensing models show evidence of improved prospective prediction of daily depressive symptom severity beyond a simple person-specific baseline?

Additionally, the following research questions were investigated:

Research question 2 (RQ2): For patients in whom models show evidence of improvement over the person-specific baseline, which sensor-derived feature groups contribute most to the prediction of individual depressive symptom severity?

Research question 3 (RQ3): Do nonlinear models change (1) the number of patients showing evidence of improved prediction beyond baseline and (2) the relative contribution of sensor feature groups compared with linear models?

## Methods

### Study Design and Cohort

The data for the present investigation were prospectively collected as part of the MONDY exploratory n-of-1 trial (German Clinical Trials Register DRKS00032618). This study’s design involved repeated measurements of self-reported, behavioral, and physiological outcomes over 1 year (365 d) within individuals without manipulation of variables by the investigators. The work was carried out in accordance with the World Medical Association Declaration of Helsinki [25].

In total, 15 adults with recurrent major depression participated in this study and self-monitored their symptoms for up to 12 months using self-report questionnaires and smartphone and smartwatch data. Participants completed the Patient Health Questionnaire-2 (PHQ-2 [26]) every evening and a full Patient Health Questionnaire-9 [27] once per week, providing repeated self-reported measures of depressive symptom severity. MS was carried out with a Samsung Galaxy Watch 5 and the companion “iTrackDepression” smartphone app, which continuously recorded the following data modalities: telephone activity, daily prompted voice recordings (30‐60 s) in response to randomly selected questions from a predefined set of 16 prompts for acoustic analysis, step counts, wrist accelerometry, app-usage logs, message traffic, and optical heart-rate-derived HRV, as detailed in the MS section of the MONDY study protocol [24].

### Feature Extraction and Grouping

#### Daily Segmentation and Grouping of Features

To examine within-person relationships between passive sensing data and depressive symptoms, all sensor-derived features were aligned to 2 fixed daily windows relative to the evening PHQ-2 rating: the preceding night (6 PM-6 AM) and the same-day daytime period (6 AM-6 PM). This segmentation captures established diurnal patterns in behavior and physiology while ensuring temporal alignment between predictors and outcomes [28,29].

To facilitate interpretable modeling and explainability analyses, derived variables were organized into modality-specific feature groups representing literature-based clusters of behavioral or physiological information. Feature groups served as units for later permutation-based domain contribution analyses.

To reduce redundancy in the high-dimensional feature space, we applied a 2-step collinearity control procedure. First, within homogeneous feature families, we removed 1 variable from pairs with very high Spearman correlation (|ρ|≥0.90), retaining the more interpretable or statistically stable representative [30]. Second, the remaining variables underwent iterative variance inflation factor pruning using a threshold of 10 until multicollinearity was reduced or only domain-anchored features remained [31]. Domain-specific rules further avoided algebraic dependencies (eg, simultaneous inclusion of raw values, normalized variants, and ratios) and prioritized features consistent with established measurement conventions in HRV and accelerometry research [32,33]. After feature extraction, grouping, and collinearity reduction, the final configured feature set comprised 130 predictors across 12 feature groups. All retained predictors, their feature-group assignments, and the preprocessing scaler applied to each predictor are reported in Multimedia Appendix 1.

#### Outcome Variable

For the outcome variable, daily depressive symptom severity was assessed using an adapted version of the PHQ-2 [26], capturing the 2 core symptoms of depression (loss of interest or joylessness and feeling down, depressed, or hopeless). Following previously established procedures [20,34], answers were given on a visual analog scale ranging from 0‐10 with labeled end points (0=never, 10=all the time). Therefore, the outcome variable was a daily adapted PHQ-2 symptom sum score based on the 2 PHQ-2 core symptoms assessed each evening on 0‐10 visual analog scales.

#### Time Features

To account for secular trends and calendar-related effects, a baseline time-related features (eg, day of week, month of year, and elapsed time; TIME) block was included containing elapsed study time, day of week, and month of year.

#### Speech and Acoustic Features

Participants provided prompted free-speech recordings in response to up to 4 randomly selected questions each day from a predefined set of 16 prompts. No standardized text was read, and only acoustic characteristics, not speech content, were analyzed. Acoustic descriptors were extracted from the 30‐60 s evening Daily-Dialog recordings using the openSMILE toolkit [35]. The resulting speech and acoustic feature group captured key dimensions of vocal expression, including loudness and its variability (vocal energy and emphasis), perturbation measures such as jitter and shimmer (voice stability), and the first 4 mel-frequency cepstral coefficients representing the spectral envelope and timbral properties of speech. These parameters summarize prosodic, spectral, and voice-quality characteristics that have been associated with mood and suicide risk in prior work [36-41].

#### Phone Communication Features

Phone behavior was summarized in the phone communication features feature group using 4 complementary metrics computed separately for day and night windows: total call duration (interaction intensity), call frequency (activity volume), number of unique contacts (network breadth), and missed calls (responsiveness or opportunity for interaction).

#### Activity Features

Wrist accelerometer data were processed using the SciKit-Digital-Health framework [32] following standard preprocessing steps including resampling, gravity correction, and reduction to the Euclidean Norm Minus One activity signal. The resulting activity features (accelerometry-derived features) feature set was grouped into 3 domains capturing core motor correlates of depression: fragmentation (irregularity and burstiness of activity [activity fragmentation]), intensity (overall motor output and time spent in activity states [activity intensity]), and rhythmicity (circadian regularity and temporal organization of activity patterns [activity rhythmicity]).

#### App Usage and Social Interaction Features (App Traffic Features and App Usage Features)

Smartphone interaction features were derived from passive Android sensors capturing app traffic and foreground usage. Daily transmitted and received data volumes and total foreground durations were aggregated for communication and social media apps identified via Google Play metadata. These variables formed the feature groups app traffic features, app usage of communication apps, and app usage of social media apps, representing digital interaction density and active engagement with socially oriented apps. This grouping reflects evidence that app traffic volume provides an indirect proxy for digital interaction density, whereas app foreground time quantifies active engagement, and that such digital behavioral markers are associated with stress, anxiety, and depressive symptoms [42-44].

#### HRV Features

Interbeat interval series were preprocessed using NeuroKit2 for beat detection, artifact correction, and quality control [33]. From valid segments, time-domain, frequency-domain, and nonlinear HRV metrics capturing overall variability, autonomic balance, and signal complexity were derived. These variables were combined into the HRV feature group.

#### Sleep Features

Sleep duration was estimated using HRV-derived sleep staging with the deep-learning classifier wrn-gru-mesa [45]. Cleaned interbeat interval sequences were segmented into epochs and classified into sleep stages, and total daily sleep time (hours) was computed. This variable defined the sleep duration feature group representing overall nightly sleep duration.

#### Adherence Indicator Features

To capture behaviorally informative absence patterns, we derived minimal missing not at random (MNAR) indicators across sensing modalities. These daily flags captured sensor dropout, diurnal imbalance, low coverage, or consecutive absence streaks in HRV, accelerometry, calls, audio recordings, sleep estimation, and app usage streams. All adherence indicators were combined into a single adherence indicator features feature group reflecting sensor availability and digital engagement patterns.

### Data Analysis

#### Participant Exclusion Due to Insufficient Data

Participants were anonymized using single-letter identifiers (eg, A, B, and C) and excluded if their time series did not meet the minimal length needed to construct at least one temporally valid train-test split. A time-based validation scheme with a minimum 60-day training window and a subsequent 30-day test window was chosen to reflect the intended prospective prediction scenario, prevent temporal leakage, and account for the nonstationary nature of depressive symptom dynamics. Consequently, 4 of the 15 participants with fewer than 90 valid daily assessments were excluded and not used for model training. This requirement was determined by the sum of the minimum training window (60 d) and the minimum test-window constraints (30-d holdout window).

#### Data Preprocessing

All preprocessing steps were performed separately for each participant and implemented as a single preprocessing pipeline within each cross-validation fold. To preserve the temporal order of observations and prevent information leakage, every preprocessing component (eg, imputation and scaling) was fitted exclusively on the training data and subsequently applied unchanged to the corresponding test window.

Daily sensor features were first aligned to the corresponding evening PHQ-2 score, and days without a valid outcome were removed. As mobile and wearable sensor data naturally contain extreme or physiologically implausible values, often due to recording artifacts, intermittent device use, or measurement noise, outliers were identified using the Tukey rule [46]. Values lying more than 1.5 times the IQR beyond the first or third quartile were set to missing. This reduced the influence of extreme observations on subsequent modeling.

No explicit feature exclusion threshold based on missingness was applied. Missing values were imputed using multivariate imputation by chained equations [47], implemented with scikit-learn’s IterativeImputer (maximum 50 iterations, convergence tolerance of 1×10⁻²) [48]. Consequently, all participant-specific models were fitted using the same set of 130 features. A participant-level summary of feature missingness before imputation is provided in Multimedia Appendix 2. Among the 11 participants included in modeling, the median number of features exceeding 50% missingness was 0 (IQR 0.0-1.5), and the median number exceeding 70% missingness was 0 (IQR 0.0-0.5). The 50% and 70% thresholds were used descriptively and were not applied as feature-exclusion criteria. Features were retained despite high missingness because the proportion of missing data alone was not considered a sufficient criterion for exclusion. Previous work [49] demonstrated that multivariate imputation by chained equations can yield unbiased estimates even with up to 90% missingness when auxiliary information is available. To partially account for informative missingness, adherence-related indicators were deterministically derived from the original missingness patterns before imputation and subsequently included as auxiliary predictor variables; these indicators themselves were not imputed. In MS and mental health contexts, missingness is often MNAR and reflects user adherence or disengagement, making omission of such variables potentially biasing.

Continuous predictors were transformed using a 2-stage scaling process, designed both to harmonize feature distributions and to create optimal conditions for EN regularization. In the first stage, each continuous feature underwent 1 of 3 possible transformations depending on its distributional properties: right-skewed variables were stabilized using a natural logarithm transformation (log1p), variables with bounded ranges were normalized to the [0, 1] interval using minimum-maximum scaling, and approximately symmetric variables were standardized to have mean 0 and SD 1. These transformations ensured that features spanning different physical units (eg, app usage seconds, HRV indices, and accelerometer-based counts) were brought to a common numerical scale (the scaler used for each feature is described in Multimedia Appendix 1). In the second stage, all features, regardless of the transformation applied in the first stage, were again z-standardized. The second standardization step ensured centered and variance-normalized inputs for EN regularization. For consistency, the same preprocessed feature matrix was supplied to RF models.

#### Model Training and Validation

##### Prediction Models

Daily adapted PHQ-2 sum scores served as the dependent variable. Two complementary regression model types were considered: EN regression [50] and RF regression [51], following prior work by Balliu et al [4] and Price et al [52].

Model development followed a nested time-series cross-validation procedure designed to preserve temporal ordering while preventing optimistic performance estimates.

##### Outer Cross-Validation (Model Evaluation)

The outer loop implemented a repeated time-series cross-validation scheme with 10 splits and 5 repetitions. Each split trained on at least 60 consecutive days and evaluated predictions on a subsequent window of up to 30 days (minimum 14 days of valid observations). To increase robustness, the starting point of the cross-validation procedure was randomly offset within the first 20% of the time series in each repetition. This produced multiple forecast origins while maintaining chronological ordering.

Within each outer fold, the model was trained only on past observations and evaluated on future observations, approximating a prospective forecasting scenario.

##### Inner Cross-Validation (Hyperparameter Tuning)

Hyperparameters were tuned exclusively within the training portion of each outer fold using an inner time-series cross-validation procedure based on TimeSeriesSplit. For EN, the grid search explored combinations of the regularization strength α and the L1-L2 mixing parameter (see Multimedia Appendix 3). For RF, grid search was performed over tree depth, number of estimators, and minimum leaf size. Model selection in the inner loop used the coefficient of determination (*R*²) as the optimization criterion.

After hyperparameter selection, the model was refitted on the full outer training window using the selected hyperparameters and evaluated on the corresponding outer test window.

### Eligibility Analysis

To address research question 1 (RQ1) and research question 3 (RQ3), the first analytical step determined whether EN and RF models captured predictive structure beyond a trivial baseline for each participant.

Within each outer fold, predictions from the tuned EN and RF models were compared with a constant baseline model implemented as a DummyRegressor [48] that always predicted the participant’s mean PHQ-2 score observed in the training window. This simple person-specific baseline was intentionally chosen to represent prediction based on symptom history alone. Accordingly, improvement beyond this baseline indicates incremental predictive information from passive sensing, but not necessarily clinically useful prediction accuracy.

Two performance metrics were computed for each fold: (1) *R*² reflecting predictive performance relative to the test-set mean and (2) mean absolute error (MAE), representing the average absolute prediction error. Performance was expressed relative to the baseline model: Δ*R*²=*R*²_model−*R*²_baseline; ΔMAE=MAE_baseline−MAE_model. As the baseline model was fitted on the training window and evaluated on temporally subsequent test observations, its *R*² was not constrained to zero and could vary across folds, including negative values. Positive values therefore indicated improved predictive performance relative to the constant predictor.

To quantify the reliability of these improvements at the participant level, a weighted bootstrap procedure with 20,000 resamples was applied. For each participant and model type, folds were resampled with replacement and the weighted mean improvement in change in coefficient of determination relative to baseline (Δ*R*²) and change in mean absolute error relative to baseline (ΔMAE) was computed, using the number of test observations in each fold as weights. This yielded an average improvement estimate and a 1-sided *P* value. To account for multiple testing across participants and model types, *P* values were adjusted using the Benjamini-Hochberg procedure controlling the false discovery rate (FDR) at *α*=.05. Adjustment was performed separately for each performance metric (Δ*R*² and ΔMAE) across both model types. This approach was selected to limit the expected proportion of false-positive findings across the multiple participant-level comparisons while maintaining sensitivity for this exploratory analysis.

A model type was considered to significantly outperform the baseline if either Δ*R*² or ΔMAE exhibited an FDR-adjusted *P* value <.05. This inclusive criterion was chosen because the 2 metrics capture complementary aspects of predictive performance (relative model fit [Δ*R*²] and absolute prediction error), and the objective of this screening step was to identify participant-model combinations showing any evidence of predictive information beyond the person-specific baseline.

Participant-model combinations that significantly outperformed the baseline were retained for the corresponding model-specific explainability analysis. Participants for whom neither model type satisfied the eligibility criterion were classified as noneligible. Comparisons between EN and RF feature-group contributions were restricted to participants whose models were eligible under both approaches.

The nested cross-validation procedure served as the primary participant-level eligibility and model-comparison stage, providing leakage-safe and temporally prospective estimates of predictive performance relative to baseline. The subsequent 50% train-holdout split was not used as an additional confirmatory test of eligibility, but rather as a standardized framework for retuning eligible models and conducting rolling explainability analyses under a fixed prospective reference period.

### Explainability Analysis

To address RQ2 and the feature-importance component of RQ3, a rolling, time-aware permutation-importance analysis was performed for all eligible participant-model combinations.

For these analyses, an initial training window covering the first 50% of the participant’s observation period (in calendar time) was defined, with the remaining 50% reserved as a prospective holdout period. The preprocessing pipeline was fitted solely on the initial training window and applied to both training and holdout data. Hyperparameters were retuned within this initial window using time-series cross-validation and subsequently fixed for the explainability stage.

Using these fixed models, expanding rolling-origin evaluation windows were constructed across the observation period. Starting from the initial training window, the training set was extended in 30-day increments, each paired with a subsequent 30-day test window. In each fold, a new model was trained on the current training window and evaluated on the corresponding test window.

Feature relevance was assessed using block-wise permutation importance, a model-agnostic method that quantifies the deterioration in predictive performance when feature information is disrupted. To preserve temporal dependencies and short-term autocorrelation, permutations were applied within contiguous 7-day blocks rather than individual observations. For each feature, the column was permuted in 7-day blocks across 50 repetitions, and the resulting change in *R*² and MAE relative to the original model was recorded.

Relative importance scores were then computed by dividing the change in MAE by the baseline MAE of the fold (relative ΔMAE) and the change in *R*² by the absolute baseline *R*² (relative Δ*R*²). These relative metrics express the proportional degradation in predictive accuracy caused by permuting a feature and allow comparison across participants, folds, and model types. As relative Δ*R*² can become unstable when baseline *R*² is close to 0, interpretation focused primarily on relative ΔMAE.

To mitigate distortions caused by multicollinearity among individual variables, permutation importance was also computed at the feature-group level by jointly permuting all variables belonging to the same sensor or behavioral category (eg, speech and acoustic features, app usage features [APP_USAGE], and HRV). Group-level importance was calculated analogously to feature-level importance and aggregated across folds. These importance scores quantify the predictive reliance of the fitted model on a feature group under temporal perturbation and should not be interpreted as causal effects of that domain on depressive symptoms. Negative contributions, rare cases where permutation accidentally improved predictions, were truncated at zero before aggregation.

For each participant and model type, importance scores were finally aggregated across rolling windows to obtain participant-level summary estimates of feature and feature-group contributions. These participant-level importance profiles were then summarized across eligible participants to compare the distribution and rank order of behavioral and physiological information sources between EN and RF models.

This framework allowed evaluation of whether a common pattern of dominant feature groups emerged across individuals and whether nonlinear RF models exploited the available behavioral signals differently from linear EN models.

### Protocol Deviations

Although this study closely followed the preregistered methods protocol [53], several methodological refinements were introduced to enhance statistical validity and interpretability. The first adjustment concerned the definition of eligibility for downstream explainability analyses. In the preregistered plan, participants were to be considered eligible for further analysis only if their models achieved an *R*² of at least 0.30 and an MAE below 1. However, preliminary analyses revealed that no participant met both criteria, despite clear evidence of systematic within-person associations in several cases [54]. To avoid excluding all participants while maintaining rigorous evaluation, the fixed thresholds were replaced by a bootstrap-based statistical test that determined whether a model’s predictive accuracy exceeded that of a constant baseline predictor. Participants whose models failed to show a statistically significant improvement over this baseline were excluded from further modeling, whereas those with significant improvements were retained. This inferential approach allowed for individualized assessment of model performance and preserved the intent of the original research question.

The second modification involved the explainability procedure. The preregistered protocol proposed using the model-agnostic Kernel SHAP framework. During implementation, this was replaced by rolling block-wise permutation importance for both model types because Kernel SHAP relies on feature-independence assumptions that are difficult to reconcile with strongly autocorrelated behavioral data and grouped sensor features, whereas block-wise permutation importance remains model-agnostic, respects temporal structure, and scales tractably across all participants and folds.

All analyses were conducted in Python using scikit-learn [48]. The complete analysis pipeline has been made publicly available [55], which also provides the software environment and dependency specifications required to reproduce the analyses.

### Ethical Considerations

Ethical approval has been granted by the Ethics Committee of the Department of Medicine at Goethe University Frankfurt, Germany (date: August 24, 2023, reference number: 2023‐1309).

Participants were recruited at the Department of Psychiatry, Psychosomatic Medicine and Psychotherapy, University Hospital of Goethe University Frankfurt, Germany. Treating physicians and psychologists were informed about this study and invited eligible patients with affective disorders to participate. Interested patients provided their contact details and agreed to be contacted by this study’s center. All participants provided written informed consent before inclusion in this study.

The legal basis for data processing was voluntary informed consent in accordance with Article 6 (1)(a) of the General Data Protection Regulation (GDPR). Data were collected and processed only after participants had provided written informed consent following detailed study and data protection information. Additional permissions for access to individual smartphone sensors were obtained within the iTrackDepression app.

## Results

### RQ1: How Many Patients Show Predictable Depression Patterns?

Table 1 summarizes participant-level improvements in predictive accuracy for the EN models, expressed as the mean change in explained variance (Δ*R*²) and the mean reduction in prediction error (ΔMAE) compared to a constant baseline predictor.

Among the evaluable participants, 7 participants showed significant improvement in Δ*R*² and 8 participants in ΔMAE; 7 participants satisfied both criteria and 1 participant satisfied only the ΔMAE criterion. Participants A, C, F, G, I, J, K, and O showed significant gains in either *R*² or MAE (all FDR-adjusted *P* values <.05), classifying them as eligible participants under the revised inferential definition. Conversely, participants H, M, and N did not show significant improvement in either performance measure and were classified as ineligible participants. Participant I exhibited substantially larger Δ*R*² values than the remaining participants. These values resulted from comparison against a participant-specific baseline model with strongly negative prospective *R*² values rather than from explained variance exceeding its theoretical upper bound. Accordingly, the reported Δ*R*² values should be interpreted as large improvements relative to the baseline predictor rather than as conventional measures of explained variance. As shown in Multimedia Appendix 4, these improvements were observed consistently across the rolling evaluation folds rather than being driven by a small number of extreme observations. The fold-wise MAE plots likewise demonstrate consistent reductions in prediction error relative to the baseline, indicating that the large Δ*R*² values reflect sustained improvement over a poorly performing baseline rather than isolated outliers or numerical artifacts. The corresponding fold-wise eligibility analysis is provided in Multimedia Appendix 4.

Overall, 8 of the 11 (73%) participants with sufficient data (95% Wilson CI 43%‐90%) showed evidence of improved predictive performance of the EN models over the baseline model. This indicates that, for several individuals, smartphone and wearable features added incremental predictive information beyond the person-specific baseline.

**Table 1.** Participant-level elastic net performance relative to a constant baseline predictor.

Participant ID	Mean Δ*R*² (SD)	Δ*R*² *P* value (FDR^a^-adjusted)	Mean ΔMAE^b^ (SD)	ΔMAE *P* value (FDR-adjusted)
A	0.549 (0.986)	.001	0.147 (0.408)	.03
C	1.079 (3.577)	.002	0.096 (0.214)	.001
F	2.645 (4.309)	<.001	0.763 (0.848)	<.001
G	0.039 (0.119)	.02	0.047 (0.110)	.002
H	−0.049 (0.158)	>.99	−0.046 (0.115)	>.99
I	15.416 (17.114)	<.001	1.232 (0.653)	<.001
J	0.058 (0.094)	<.001	0.084 (0.102)	<.001
K	1.165 (1.510)	<.001	0.217 (0.293)	<.001
M	0.053 (0.221)	.34	0.043 (0.134)	.23
N	−0.071 (0.221)	>.99	−0.085 (0.276)	>.99
O	0.045 (0.307)	.24	0.072 (0.212)	.01

a FDR: false discovery rate.

b MAE: mean absolute error.

### RQ2: Which Sensor Domains Contribute Most to Individual-Level Predictions?

**Figure 1.**
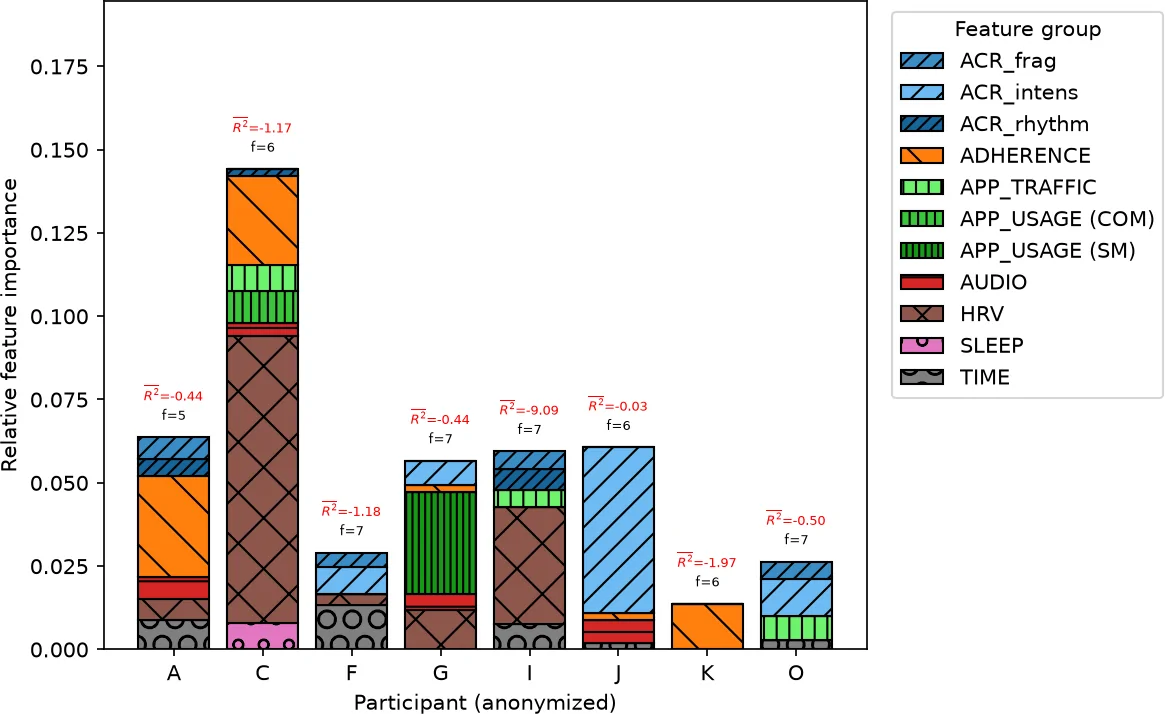
Relative contribution of feature groups in elastic net models across participants. Feature groups are color-coded according to the legend. Stacked bars show positive relative feature importance based on Δmean absolute error (MAE) for each participant after thresholding. Participants are anonymized by letters along the x-axis. The height of each stacked segment represents the mean relative MAE contribution of the respective feature group across rolling evaluation folds. Annotations above the bars indicate the mean *R*² of the participant-specific model and the number of evaluation folds (f); negative mean *R*² values are shown in red. ACR_frag: activity fragmentation; ACR_intens: activity intensity; ACR_rhythm: activity rhythmicity; ADHERENCE: adherence indicator features; APP_TRAFFIC: app traffic features; APP_USAGE (COM): app usage (communication apps); APP_USAGE (SM): app usage (social media apps); AUDIO: speech and acoustic features; HRV: heart rate variability; SLEEP: sleep duration feature group; TIME: time-related features (eg, day of week, month of year, and elapsed time).

Permutation-based explainability analyses revealed substantial variation in the relative importance of different feature groups across participants. Figure 1 provides a participant-level stacked bar visualization of relative feature-group contributions, where each bar represents 1 participant and each color segment corresponds to a feature group.

Relative contributions reflect the proportion of prediction degradation attributable to permuting a feature group, expressed using the relative ΔMAE metric, which normalizes contributions within folds and across participants.

Across participants, the permutation-based feature-importance profiles demonstrated substantial heterogeneity in the relative contributions of different feature groups, underscoring the individualized nature of the learned associations. As shown in Figure 1, no single feature group was consistently dominant across all participants. Instead, the relative influence of sensor modalities varied markedly from person to person. For example, participant G showed comparatively strong contributions from APP_USAGE features, whereas participants C and I exhibited greater influence from HRV-related features. In participants A, C, G, and K, adherence-related features contributed noticeably to model performance. In addition, accelerometry-derived activity regularity measures were among the more influential features for all participants except K. A subset of participants (A, C, G, and J) also showed meaningful contributions from audio features, although these patterns appeared idiosyncratic rather than systematic.

Fold-wise permutation importance profiles for individual participants, illustrating temporal variability in feature-group contributions across expanding windows, are shown for EN models in Multimedia Appendix 4.

### RQ3: Do Nonlinear Models Improve Prediction and Change Domain Importance?

#### Model Performance of RF Models Relative to Baseline

RF models exhibited a somewhat different performance profile compared to EN models, consistent with the possibility that nonlinear relationships were relevant for some participants. Table 2 reports participant-level improvements over the baseline mean predictor. As with EN models, participants were considered eligible when performance gains were statistically significant.

A total of 6 participants showed significant improvement in Δ*R*² and 8 participants in ΔMAE; 6 participants satisfied both criteria and 2 participants satisfied only the ΔMAE criterion. Participants A, F, G, I, J, K, M, and O showed statistically significant improvements in at least 1 of the 2 performance metrics, classifying them as eligible under the revised inferential criteria. For most of these participants, improvements were observed in both explained variance (Δ*R*²) and prediction error (ΔMAE), with FDR-adjusted *P* values below .05 indicating consistently positive out-of-sample gains across cross-validation folds. Participants G and M were considered eligible based on a significant reduction in MAE despite a nonsignificant Δ*R*². In contrast, participants C, H, and N did not exhibit improvements on either metric, showing no evidence of predictive structure detectable by the RF model.

**Table 2.** Participant-level random forest performance relative to constant baseline predictor.

Participant ID	Mean Δ*R*² (SD)	Δ*R*² *P* value (FDR^a^-adjusted)	Mean ΔMAE^b^ (SD)	ΔMAE *P* value (FDR-adjusted)
A	0.624 (0.838)	<.001	0.187 (0.345)	.001
C	0.529 (2.741)	.16	0.092 (0.600)	.20
F	2.615 (4.158)	<.001	0.746 (0.776)	<.001
G	0.033 (0.260)	.25	0.120 (0.257)	.002
H	−0.121 (0.505)	>.99	−0.092 (0.469)	>.99
I	14.012 (16.403)	<.001	1.069 (0.551)	<.001
J	0.099 (0.160)	<.001	0.206 (0.196)	<.001
K	1.913 (2.213)	<.001	0.392 (0.413)	<.001
M	0.257 (0.505)	.12	0.241 (0.208)	.001
N	−0.270 (0.556)	>.99	−0.325 (0.792)	>.99
O	0.278 (0.443)	<.001	0.250 (0.324)	<.001

a FDR: false discovery rate.

b MAE: mean absolute error.

Overall, 8 of 11 (73%) participants with sufficient data (95% CI 43%‐90%) demonstrated statistically reliable improvements over baseline when modeled with RF models, consistent with the overall rate of eligible participants observed for the EN models. However, the sets of eligible participants were not identical across modeling approaches, indicating that the 2 model classes captured predictive signal in partly different individuals. This finding suggests that both linear and nonlinear approaches were able to detect incremental within-person predictive signal in some individuals, although the magnitude and composition of these gains differed across participants.

Detailed per-participant results underlying the eligibility analysis, including fold-wise performance of EN and RF models relative to the baseline and the corresponding bootstrap-based improvements (Δ*R*² and ΔMAE), are provided in Multimedia Appendix 4.

#### Relative Contribution of Feature Groups in RF Models

**Figure 2.**
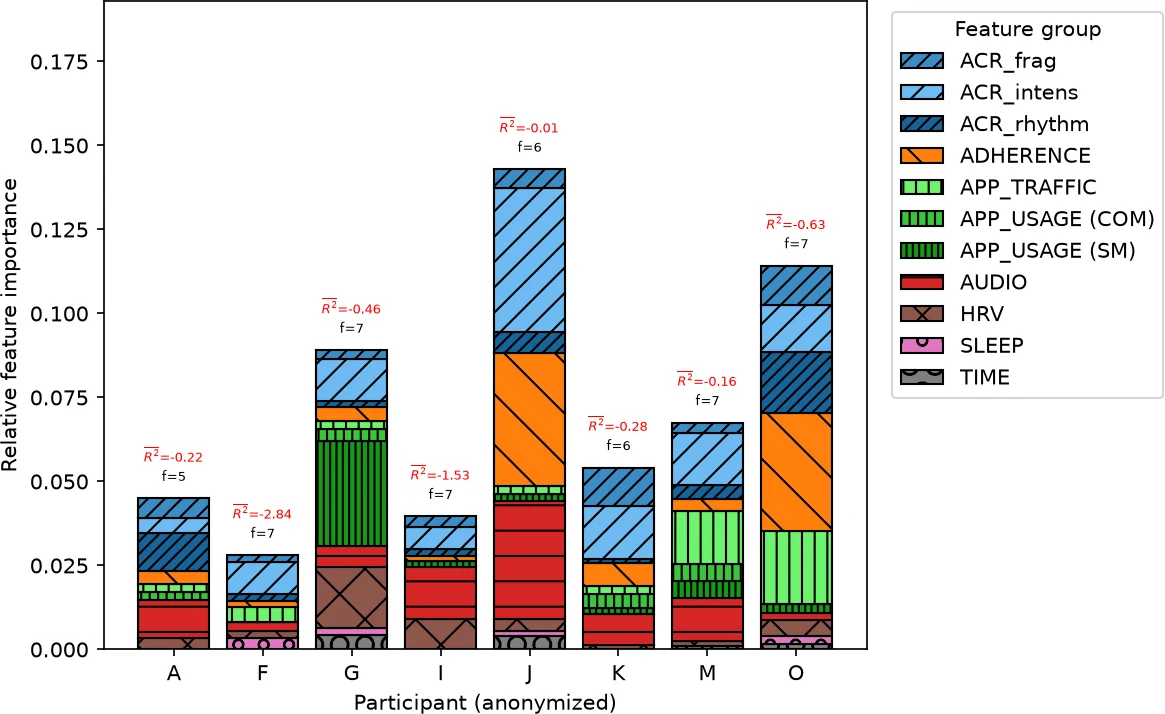
Relative contribution of feature groups in random forest models across participants. Feature groups are color-coded according to the legend. Stacked bars show positive relative feature importance based on Δmean absolute error (MAE) for each participant after thresholding. Participants are anonymized by letters along the x-axis. The height of each stacked segment represents the mean relative MAE contribution of the respective feature group across rolling evaluation folds. Annotations above the bars indicate the mean *R*² of the participant-specific model and the number of evaluation folds (f); negative mean *R*² values are shown in red. ACR_frag: activity fragmentation; ACR_intens: activity intensity; ACR_rhythm: activity rhythmicity; ADHERENCE: adherence indicator features; APP_TRAFFIC: app traffic features; APP_USAGE (COM): app usage (communication apps); APP_USAGE (SM): app usage (social media apps); AUDIO: speech and acoustic features; HRV: heart rate variability; SLEEP: sleep duration feature group; TIME: time-related features (eg, day of week, month of year, and elapsed time).

Permutation-based feature-importance patterns for the RF did not indicate a uniform increase in feature-group contributions across participants, but rather revealed pronounced interindividual differences. As shown in Figure 2, for specific participants (notably K and O), the RF models were able to extract incremental predictive information from multiple feature domains. In contrast, the corresponding EN models for these participants showed very limited feature-group contributions, suggesting that the linear models were unable to leverage the available signals effectively in these cases. For other participants, however, feature-group contributions were comparable between model types. Despite this heterogeneous pattern, a paired Wilcoxon signed-rank test showed a statistically detectable difference in aggregated feature-group importance between RF and EN (*P*=.03). To assess the influence of participant I, the aggregate comparison was repeated after excluding this participant. The resulting feature-group importance profiles remained qualitatively similar, and the paired Wilcoxon signed-rank test likewise remained significant (*P*=.005), indicating that the observed aggregate difference was not driven by this participant alone (Multimedia Appendix 5).

Across participants, feature groups related to app usage, activity regularity (activity features [accelerometry-derived features]), and audio characteristics frequently accounted for a substantial proportion of the predictive signal (Figure 2). At the same time, nearly all feature groups, including HRV, adherence, temporal, and sleep features, showed notable permutation importance in at least some individuals, highlighting the broad range of behavioral information captured by the models. For example, participants A, F, G, I, J, K, M, and O showed marked contributions from app-usage and activity-related features, whereas sleep features were particularly influential for participants F, G, J, and O. Overall, no single feature group consistently dominated across participants, reflecting substantial interindividual heterogeneity in the learned feature-importance profiles.

Corresponding fold-wise permutation importance plots for RF models at the participant level are provided in Multimedia Appendix 4.

#### Comparison of Feature-Group Contributions Between Model Types

**Figure 3.**
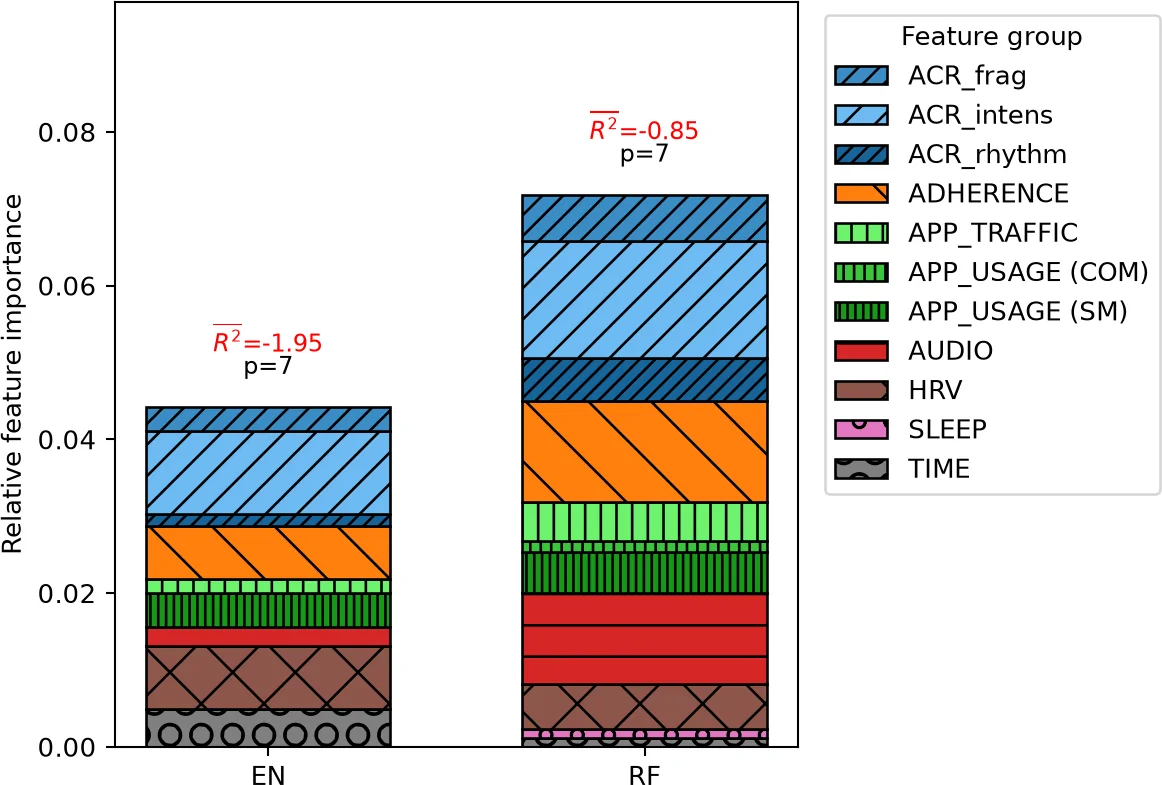
Comparison of aggregated feature-group contributions between EN and RF models. The feature groups are color-encoded according to the legend on the right. Stacked bars represent the positive relative feature importance (Δmean absolute error [MAE]) aggregated over participants, with each bar showing the combined contribution of all feature groups retained after thresholding. The height of each stacked segment reflects the mean relative MAE contribution of that feature group over participants. Annotations above each bar indicate the average model *R*² across all test folds and included participants, together with the number of participants contributing to the aggregate (p). ACR_frag: activity fragmentation; ACR_intens: activity intensity; ACR_rhythm: activity rhythmicity; ADHERENCE: adherence indicator features; APP_TRAFFIC: app traffic features; APP_USAGE (COM): app usage (communication apps); APP_USAGE (SM): app usage (social media apps); AUDIO: speech and acoustic features; EN: elastic net; HRV: heart rate variability; RF: random forest; SLEEP: sleep duration feature group; TIME: time-related features (eg, day of week, month of year, and elapsed time).

Figure 3 presents an aggregated comparison of relative feature-group contributions for the EN and RF models. Each stacked bar summarizes the mean importance values across participants who showed significant predictive improvements in both model types. As this comparison was restricted to participants showing significant predictive improvement with both model types, the aggregated comparison was based on only 7 individuals and should therefore be interpreted as exploratory.

Feature-group permutation importance showed broadly similar contribution patterns across EN and RF models (Figure 3). Accelerometry-derived features (activity fragmentation, activity intensity, and activity rhythmicity) contributed most to prediction in both modeling approaches. Smartphone interaction features (APP_USAGE and app traffic features) showed moderate contributions, whereas physiological features such as sleep duration feature group were generally less influential. A notable difference between models was the greater relative importance of calendar-based covariates (TIME) in the EN models, whereas RF models generally showed larger normalized permutation-importance values across the remaining feature groups, suggesting that the nonlinear models were able to leverage predictive information more effectively across multiple sensing domains.

## Discussion

### Principal Findings

This study evaluated whether multimodal smartphone and wearable data provide predictive information beyond a simple person-specific baseline under strictly prospective, time-aware evaluation and, where predictive value was observed, which sensor domains contributed most to these predictions. In line with the primary objective (RQ1), participant-specific models identified incremental predictive signal in a subset of individuals, with 8 of 11 (73%) participants (95% Wilson CI 43%‐90%) showing significant improvement over baseline for each model type. Although the overall number of participants showing significant improvement was identical, the sets of eligible participants were not completely overlapping, further emphasizing the participant-specific nature of predictive performance. Consistent with RQ2, no single sensor domain consistently dominated prediction across participants; instead, feature-group importance profiles were highly heterogeneous. Regarding RQ3, RF models exhibited statistically greater aggregated feature-group contributions than EN models, suggesting differences in how the 2 model classes used the available sensor information during prediction. However, this did not translate into a higher overall proportion of participants showing significant improvement over the person-specific baseline, indicating that nonlinear models appear to exploit behavioral information differently rather than uniformly improving predictive performance. Together, these findings indicate that both predictive performance and the behavioral domains underlying prediction are strongly participant-specific.

### Interpretation of the Main Findings

This study found that multimodal smartphone and wearable data provided incremental predictive information beyond a simple person-specific baseline for a majority of participants. Approximately three-quarters of participants showed statistically significant improvement over baseline for both EN and RF models. Although these proportions suggest that participant-specific predictive signal was present in many individuals, the CIs remain wide because of the limited number of evaluable participants. Moreover, predictive performance varied considerably across participants, indicating that both the strength and nature of the detectable signal were highly individualized. The wide Wilson CIs further emphasize that the observed proportion of participants showing predictive improvement should be interpreted cautiously until replicated in larger cohorts.

Importantly, improvement over a simple person-specific baseline should not be equated with clinically useful prediction accuracy. Likewise, statistically significant improvements over baseline should be interpreted alongside their effect magnitude, as some participant-model combinations exhibited only small absolute reductions in prediction error despite meeting the predefined statistical criterion. Accordingly, the present findings support the presence of incremental predictive signal in some individuals rather than establishing passive sensing as a reliable stand-alone tool for symptom monitoring. Consistent with this idiographic perspective, by focusing on individual time series rather than group-level averages (eg, Zhang et al [56]), the present study further contributes to the growing literature on idiographic MS in depression.

The observation that predictive performance was highly individualized is broadly consistent with previous personalized mobile-sensing studies, although important methodological differences should be considered when interpreting the findings. Balliu et al [4] developed participant-specific EN models to predict interpolated Computer Adaptive Test Depression Inventory scores and evaluated performance using mean absolute percentage error and *R*². Price et al [52] used a stacked ensemble incorporating RFs to predict 12-month Patient Health Questionnaire-9 symptom variability and reported correlation coefficients together with normalized MAE. These substantial differences in prediction targets, outcome measures, feature representations, and evaluation procedures preclude direct numerical comparison. At the same time, they reinforce observations from recent reviews highlighting considerable methodological heterogeneity across mobile-sensing studies [12-14] and underline the importance of rigorous prospective evaluation and model-agnostic, domain-level explainability for improving comparability between studies. Against this background, this study extends previous work by combining strictly prospective evaluation with comparison against a person-specific baseline and model-agnostic feature-group explainability, thereby contributing a structured framework for evaluating individualized mobile-sensing models under prospective, time-aware conditions.

The explainability analyses further support the interpretation that predictive relationships are highly individualized. No single sensor domain consistently dominated prediction across participants, and feature-group importance profiles differed markedly between individuals. Although accelerometry, smartphone interaction, HRV, adherence indicators, and audio features contributed meaningfully for some participants, no common sensing profile emerged. Instead, different participants relied on different combinations of behavioral domains. This pattern is consistent with previous evidence that depression follows highly individualized behavioral trajectories and evolving within-person dynamics rather than stable group-level patterns [17,20]. The comparatively greater contribution of the TIME feature group in EN models likely reflects the ability of linear models to exploit approximately monotonic temporal trends over the observation period. In particular, elapsed study time likely captures gradual longitudinal changes in depressive symptom severity rather than psychologically meaningful behavioral variation, although day of week and month of year may additionally account for calendar-related temporal structure. Consequently, the TIME feature group should be interpreted as representing temporal adjustment within the observation period rather than a passive sensing modality. As permutation importance reflects model reliance rather than causation, the identified feature groups should be interpreted as predictive correlates of symptom variation rather than mechanistic drivers of depression.

Finally, the comparison between EN and RF suggests that the 2 modeling approaches may exploit available sensor information differently rather than consistently improving prediction. RF models showed larger aggregated permutation-importance values and, for some participants, relied on a broader range of domains. Nevertheless, RF did not identify predictive improvement in more participants than EN. As the aggregate comparison was based on only 7 participants with eligible models under both approaches, these findings should be interpreted cautiously. Given the exploratory comparison involving 7 participants, these findings do not establish a general advantage of nonlinear modeling but suggest that model suitability may depend on the individual symptom-behavior relationship.

### Limitations

Several limitations should be considered when interpreting the present findings.

First, participant-specific modeling of 130 predictors from relatively short individual time series creates a risk of overfitting, despite nested time-series validation and regularization. In addition, unmeasured time-varying factors such as treatment changes, life events, or contextual changes may have influenced both sensor features and symptom ratings. Second, as a proof-of-concept study on individualized, long-term depression monitoring, the current work is based on a small sample. Given the heterogeneity of depression, the symptoms and patterns identified here may not represent the broader population of patients with depressive disorders. Consequently, the generalizability of these findings is limited and will require validation in larger samples.

Third, although the target variable was assessed daily, the rolling evaluation and explainability analyses were based on 30-day test windows and 30-day training extensions. This design supports stable prospective assessment but may smooth over shorter-lived day-to-day fluctuations and transient temporal relationships that could also be clinically relevant. Consequently, short-term temporal relationships and how they may fluctuate across days remain unexamined. Future work should build on the present framework by investigating prediction errors across different levels of symptom severity, symptom transitions, and phases of longitudinal follow-up. Such analyses could help determine under which conditions passive sensing models provide reliable predictions and where their predictive performance deteriorates, thereby improving the clinical interpretability of individualized mobile-sensing models.

Fourth, although each participant contributed intensive repeated measurements, the number of independent individuals available for aggregate inference was only 11 participants, and only 7 participants contributed to the paired model-class comparison. Consequently, statistical power for participant-level inference remained limited despite the large number of repeated measurements. In addition, participant-level inference involved multiple comparisons across participants, model classes, and performance metrics. To reduce the expected proportion of false-positive findings, bootstrap-based inference was combined with Benjamini-Hochberg FDR adjustment. While this represents an appropriate strategy for exploratory analyses, it cannot fully compensate for the limited number of independent participants. Consequently, both false-positive and false-negative findings remain possible, and the present results should be interpreted as hypothesis-generating until replicated in larger independent cohorts. In addition, although participant I exhibited markedly larger improvements relative to the baseline model than the remaining participants, a sensitivity analysis excluding this participant yielded qualitatively unchanged aggregate results, supporting the robustness of the principal conclusions.

Finally, although adherence-related indicators derived from the original sensor availability patterns were included as auxiliary predictors to partially capture informative missingness, they cannot fully account for MNAR mechanisms. Consequently, if missingness depended on unobserved factors beyond those reflected by the adherence indicators, some bias in the imputed predictor values may remain.

### Conclusions

This study demonstrates that multimodal smartphone and wearable data can provide incremental predictive information beyond a simple person-specific baseline for daily depressive symptom severity in a subset of individuals when evaluated under strictly prospective, time-aware conditions. However, predictive performance remained highly participant-specific, and the observed improvements alone do not support immediate clinical application.

These findings suggest that future passive-sensing approaches for depression are unlikely to benefit from a universal sensing profile or a single modeling strategy. Instead, predictive modeling and interpretation may need to be individualized, reflecting the heterogeneous nature of depressive symptom dynamics. More broadly, these findings suggest that previously reported associations in mobile-sensing research, often derived from heterogeneous study designs and feature representations, may not consistently translate into prospective prediction. Future research may therefore benefit from evaluation frameworks that assess predictive signal under rigorous prospective, individual-level conditions while considering individualized combinations of behavioral domains rather than assuming a universal sensing profile.

## Supplementary material

10.2196/99204 Multimedia Appendix 1 Overview of all features used, their feature-group assignments, and the preprocessing scaler applied to each feature.

10.2196/99204 Multimedia Appendix 2 Table of feature missingness per subject.

10.2196/99204 Multimedia Appendix 3 Table of hyperparameter grids used for elastic net and random forest models.

10.2196/99204 Multimedia Appendix 4 Per-participant plots of eligibility and explainability results.

10.2196/99204 Multimedia Appendix 5 Sensitivity analysis of aggregated feature-group contributions after exclusion of participant I.

## Abbreviations

APP_USAGE: app usage features
EN: elastic net
FDR: false discovery rate
GDPR: General Data Protection Regulation
HRV: heart rate variability
MAE: mean absolute error
MNAR: missing not at random
MONDY: secure and open platform for AI-based health care apps
MS: mobile sensing
PHQ-2: Patient Health Questionnaire 2
RF: random forest
RQ1: research question 1
RQ2: research question 2
RQ3: research question 3
*R*²: coefficient of determination
SHAP: Shapley Additive Explanations
TIME: time-related features (eg, day of week, month of year, and elapsed time)
ΔMAE: change in mean absolute error relative to baseline
Δ*R*²: change in coefficient of determination relative to baseline
