# Supplementary material for: Individual-Level Modeling of Depressive Symptom Severity Using Smartphone and Wearable Data: Time-Aware 1-Year Study With Feature-Group Contributions
Source: JMIR Form Res. 2026 Aug 25;10:e99204. doi: 10.2196/99204 (PMC13507524; doi:10.2196/99204)
Supplement: Supplementary file 1 [file formative-v10-e99204-s001.docx]

| **Feature** | **Feature Group** | **Scaler** |
| --- | --- | --- |
| wake MVPA power law distribution_day | Activity | zscore |
| wake MVPA power law distribution_night | Activity | zscore |
| wake SLPA power law distribution_day | Activity | zscore |
| wake SLPA power law distribution_night | Activity | zscore |
| wake sed power law distribution_day | Activity | zscore |
| wake sed power law distribution_night | Activity | zscore |
| steps_sum_rq1_day | Activity | log1p |
| steps_sum_rq1_night | Activity | log1p |
| total_activity_min_day | Activity | zscore |
| total_activity_min_night | Activity | zscore |
| wake duration equal avg duration [min]_day | Activity | zscore |
| wake duration equal avg duration [min]_night | Activity | zscore |
| wake light 60s epoch [min]_day | Activity | zscore |
| wake light 60s epoch [min]_night | Activity | zscore |
| wake mod 60s epoch [min]_day | Activity | zscore |
| wake mod 60s epoch [min]_night | Activity | zscore |
| wake vig 60s epoch [min]_day | Activity | zscore |
| wake vig 60s epoch [min]_night | Activity | zscore |
| wake 60min power spectral sum_day | Activity | log1p |
| wake 60min power spectral sum_night | Activity | log1p |
| wake 60min sample entropy_day | Activity | zscore |
| wake 60min sample entropy_night | Activity | zscore |
| wake 60min signal entropy_day | Activity | zscore |
| wake 60min signal entropy_night | Activity | zscore |
| wake dfa activity balance index_day | Activity | minmax |
| wake dfa activity balance index_night | Activity | minmax |
| wake dfa alpha_day | Activity | zscore |
| wake dfa alpha_night | Activity | zscore |
| wake ig r-squared_day | Activity | minmax |
| wake ig r-squared_night | Activity | minmax |
| wake intensity gradient_day | Activity | zscore |
| wake intensity gradient_night | Activity | zscore |
| MNAR_ACR_diurnal_one_sided_day | ADHERENCE |  |
| MNAR_CALLS_diurnal_one_sided_day | ADHERENCE |  |
| MNAR_CALLS_zero_day_flag | ADHERENCE |  |
| MNAR_CALLS_zero_streak_len | ADHERENCE |  |
| MNAR_COM_TRAFFIC_diurnal_one_sided_day | ADHERENCE |  |
| MNAR_COM_TRAFFIC_zero_day_flag | ADHERENCE |  |
| MNAR_COM_TRAFFIC_zero_streak_len | ADHERENCE |  |
| MNAR_COM_USAGE_diurnal_one_sided_day | ADHERENCE |  |
| MNAR_COM_USAGE_zero_day_flag | ADHERENCE |  |
| MNAR_COM_USAGE_zero_streak_len | ADHERENCE |  |
| MNAR_DIALOG_zero_day_flag | ADHERENCE |  |
| MNAR_DIALOG_zero_streak_len | ADHERENCE |  |
| MNAR_HRV_diurnal_one_sided_day | ADHERENCE |  |
| MNAR_HRV_low_cov_day_flag | ADHERENCE |  |
| MNAR_HRV_total10h_flag | ADHERENCE |  |
| MNAR_HRV_zero_day_flag | ADHERENCE |  |
| MNAR_HRV_zero_streak_len | ADHERENCE |  |
| MNAR_SLEEP_diurnal_one_sided_night | ADHERENCE |  |
| MNAR_SLEEP_low_cov_day_flag | ADHERENCE |  |
| MNAR_SLEEP_zero_day_flag | ADHERENCE |  |
| MNAR_SLEEP_zero_streak_len | ADHERENCE |  |
| MNAR_SM_TRAFFIC_diurnal_one_sided_day | ADHERENCE |  |
| MNAR_SM_TRAFFIC_zero_day_flag | ADHERENCE |  |
| MNAR_SM_TRAFFIC_zero_streak_len | ADHERENCE |  |
| MNAR_SM_USAGE_diurnal_one_sided_day | ADHERENCE |  |
| MNAR_SM_USAGE_zero_day_flag | ADHERENCE |  |
| MNAR_SM_USAGE_zero_streak_len | ADHERENCE |  |
| total_com_rx_day | APP_TRAFFIC | log1p |
| total_com_rx_night | APP_TRAFFIC | log1p |
| total_com_tx_day | APP_TRAFFIC | log1p |
| total_com_tx_night | APP_TRAFFIC | log1p |
| total_sm_rx_day | APP_TRAFFIC | log1p |
| total_sm_rx_night | APP_TRAFFIC | log1p |
| total_sm_tx_day | APP_TRAFFIC | log1p |
| total_sm_tx_night | APP_TRAFFIC | log1p |
| total_com_app_usage_day_h | APP_USAGE (COM) | log1p |
| total_com_app_usage_night_h | APP_USAGE (COM) | log1p |
| total_sm_app_usage_day_h | APP_USAGE (SM) | log1p |
| total_sm_app_usage_night_h | APP_USAGE (SM) | log1p |
| F0semitoneFrom27.5Hz_sma3nz_amean | AUDIO | zscore |
| F0semitoneFrom27.5Hz_sma3nz_stddevNorm | AUDIO | zscore |
| jitterLocal_sma3nz_amean | AUDIO | zscore |
| loudness_sma3_amean | AUDIO | zscore |
| loudness_sma3_stddevNorm | AUDIO | zscore |
| mfcc1V_sma3nz_amean | AUDIO | zscore |
| mfcc2V_sma3nz_amean | AUDIO | zscore |
| mfcc3V_sma3nz_amean | AUDIO | zscore |
| mfcc4V_sma3nz_amean | AUDIO | zscore |
| shimmerLocaldB_sma3nz_amean | AUDIO | zscore |
| nbr_missed_calls_day | CALLS | zscore |
| nbr_missed_calls_night | CALLS | zscore |
| number_of_contacts_day | CALLS | zscore |
| number_of_contacts_night | CALLS | zscore |
| total_calling_frequency_day | CALLS | zscore |
| total_calling_frequency_night | CALLS | zscore |
| total_duration_calls_day | CALLS | zscore |
| total_duration_calls_night | CALLS | zscore |
| HRV_LF_rq1_day | HRV | log1p |
| HRV_LF_rq1_night | HRV | log1p |
| HRV_LFn_rq1_day | HRV | minmax |
| HRV_LFn_rq1_night | HRV | minmax |
| HRV_MadNN_rq1_day | HRV | zscore |
| HRV_MadNN_rq1_night | HRV | zscore |
| HRV_MedianNN_rq1_day | HRV | zscore |
| HRV_MedianNN_rq1_night | HRV | zscore |
| HRV_SD2_rq1_day | HRV | zscore |
| HRV_SD2_rq1_night | HRV | zscore |
| HRV_SDNN_rq1_day | HRV | zscore |
| HRV_SDNN_rq1_night | HRV | zscore |
| HRV_SDRMSSD_rq1_day | HRV | zscore |
| HRV_SDRMSSD_rq1_night | HRV | zscore |
| HRV_SampEn_rq1_day | HRV | zscore |
| HRV_SampEn_rq1_night | HRV | zscore |
| HRV_ULF_rq1_day | HRV | log1p |
| HRV_ULF_rq1_night | HRV | log1p |
| HRV_VHF_rq1_day | HRV | log1p |
| HRV_VHF_rq1_night | HRV | log1p |
| number_used_ibi_datapoints_rq1_day | HRV |  |
| number_used_ibi_datapoints_rq1_night | HRV |  |
| daily_sleep_duration | SLEEP | zscore |
| last_night_sleep_duration | SLEEP | zscore |
| day_since_start | TIME | minmax |
| day_of_week | TIME | minmax |
| month_of_year | TIME | minmax |
