# Supplementary material for: Individual-Level Modeling of Depressive Symptom Severity Using Smartphone and Wearable Data: Time-Aware 1-Year Study With Feature-Group Contributions
Source: JMIR Form Res. 2026 Aug 25;10:e99204. doi: 10.2196/99204 (PMC13507524; doi:10.2196/99204)
Supplement: Supplementary file 2 [file formative-v10-e99204-s002.docx]

| Participant | Days with outcome | Configured predictors | Predictors >50% missing | Predictors >70% missing | Median feature missingness (%) | Maximum feature missingness (%) |
| --- | --- | --- | --- | --- | --- | --- |
| A | 236 | 130 | 8 | 8 | 11.4 | 100.0 |
| C | 316 | 130 | 5 | 4 | 26.6 | 100.0 |
| F | 213 | 130 | 0 | 0 | 25.8 | 46.0 |
| G | 361 | 130 | 0 | 0 | 0.3 | 17.5 |
| H | 228 | 130 | 0 | 0 | 8.3 | 32.0 |
| I | 192 | 130 | 0 | 0 | 2.1 | 27.6 |
| J | 315 | 130 | 0 | 0 | 3.5 | 42.2 |
| K | 300 | 130 | 1 | 0 | 17.0 | 53.7 |
| M | 157 | 130 | 2 | 1 | 20.4 | 76.4 |
| N | 226 | 130 | 0 | 0 | 2.2 | 36.7 |
| O | 294 | 130 | 0 | 0 | 16.7 | 46.9 |

**Note.** Missingness was calculated before imputation using participant-days with an available daily PHQ-2 outcome. The table includes all 130 configured predictors, including the three TIME features. The 50% and 70% thresholds were used descriptively and were not applied as feature-exclusion criteria. Participant-specific predictors with 100% missingness were removed before model fitting because they could not be estimated from that participant's observations.
