# Supplementary material for: Individual-Level Modeling of Depressive Symptom Severity Using Smartphone and Wearable Data: Time-Aware 1-Year Study With Feature-Group Contributions
Source: JMIR Form Res. 2026 Aug 25;10:e99204. doi: 10.2196/99204 (PMC13507524; doi:10.2196/99204)
Supplement: Supplementary file 3 [file formative-v10-e99204-s003.docx]

| **Model** | **Hyperparameter** | **Grid / Values** |
| --- | --- | --- |
| **Elastic Net (EN)** | Regularization strength (α) | 60 log-spaced values from (10^-5^) to (10^0^) |
|  | Mixing parameter (l1_ratio) | {0.1, 0.5, 0.9} |
| **Random Forest (RF)** | Number of trees (n_estimators) | {100, 200, 400, 800} |
|  | Maximum tree depth (max_depth) | {4, 6, 8, 10, 15, 20, None} |
|  | Minimum samples per leaf (min_samples_leaf) | {1, 2, 4, 6, 8} |
