# Supplementary material for: Individual-Level Modeling of Depressive Symptom Severity Using Smartphone and Wearable Data: Time-Aware 1-Year Study With Feature-Group Contributions
Source: JMIR Form Res. 2026 Aug 25;10:e99204. doi: 10.2196/99204 (PMC13507524; doi:10.2196/99204)
Supplement: Supplementary file 4 [file formative-v10-e99204-s004.docx]

# Per-Participant Results

## Participant A

**Figure S1.** Eligibility analysis using repeated time-series cross-validation. Top panels show the elastic net model (orange) and constant baseline model (grey) evaluated across all repeated cross-validation folds for participant A. Left: coefficient of determination (R²) per fold. Right: mean absolute error (MAE) per fold. Bottom panels show performance improvements relative to the baseline: left, ΔR² = R²(EN) − R²(Dummy); right, ΔMAE = MAE(Dummy) − MAE(EN). Positive values indicate better performance than the baseline model. Fold-wise improvements serve as the basis for the bootstrap significance test that determines eligibility status.

**Figure S2.** Eligibility analysis using repeated time-series cross-validation. Top panels show the random forest model (red) and constant baseline model (grey) evaluated across all repeated cross-validation folds for participant A. Left: coefficient of determination (R²) per fold. Right: mean absolute error (MAE) per fold. Bottom panels show performance improvements relative to the baseline: left, ΔR² = R²(EN) − R²(Dummy); right, ΔMAE = MAE(Dummy) − MAE(EN). Positive values indicate better performance than the baseline model. Fold-wise improvements serve as the basis for the bootstrap significance test that determines eligibility status.

**Figure S3.** Fold-wise permutation importance for elastic net in participant A. Relative feature importance across rolling time-based folds for feature groups. Bars represent the proportional performance degradation (relative ΔMAE) caused by permuting each feature group within 7-day blocks in the corresponding fold. Labels on the top of the bars show the R² of the elastic net model calculated on the test observations for each fold (red if negative), and n indicates the number of test observations in that fold. Higher bars indicate greater relevance of the permuted feature group for predicting daily PHQ-2 scores in that specific temporal window.

**Figure S4.** Fold-wise permutation importance for random forest in participant A. Relative feature importance across rolling time-based folds for feature groups. Bars represent the proportional performance degradation (relative ΔMAE) caused by permuting each feature group within 7-day blocks in the corresponding fold. Labels on the top of the bars show the R² of the elastic net model calculated on the test observations for each fold (red if negative), and n indicates the number of test observations in that fold. Higher bars indicate greater relevance of the permuted feature group for predicting daily PHQ-2 scores in that specific temporal window.

## Participant C

**Figure S5.** Eligibility analysis using repeated time-series cross-validation. Top panels show the elastic net model (orange) and constant baseline model (grey) evaluated across all repeated cross-validation folds for participant C. Left: coefficient of determination (R²) per fold. Right: mean absolute error (MAE) per fold. Bottom panels show performance improvements relative to the baseline: left, ΔR² = R²(EN) − R²(Dummy); right, ΔMAE = MAE(Dummy) − MAE(EN). Positive values indicate better performance than the baseline model. Fold-wise improvements serve as the basis for the bootstrap significance test that determines eligibility status.

**Figure S6.** Eligibility analysis using repeated time-series cross-validation. Top panels show the random forest model (red) and constant baseline model (grey) evaluated across all repeated cross-validation folds for participant C. Left: coefficient of determination (R²) per fold. Right: mean absolute error (MAE) per fold. Bottom panels show performance improvements relative to the baseline: left, ΔR² = R²(EN) − R²(Dummy); right, ΔMAE = MAE(Dummy) − MAE(EN). Positive values indicate better performance than the baseline model. Fold-wise improvements serve as the basis for the bootstrap significance test that determines eligibility status.

**Figure S7.** Fold-wise permutation importance for elastic net in participant C. Relative feature importance across rolling time-based folds for feature groups. Bars represent the proportional performance degradation (relative ΔMAE) caused by permuting each feature group within 7-day blocks in the corresponding fold. Labels on the top of the bars show the R² of the elastic net model calculated on the test observations for each fold (red if negative), and n indicates the number of test observations in that fold. Higher bars indicate greater relevance of the permuted feature group for predicting daily PHQ-2 scores in that specific temporal window.

**Figure S8.** Fold-wise permutation importance for random forest in participant C. Relative feature importance across rolling time-based folds for feature groups. Bars represent the proportional performance degradation (relative ΔMAE) caused by permuting each feature group within 7-day blocks in the corresponding fold. Labels on the top of the bars show the R² of the elastic net model calculated on the test observations for each fold (red if negative), and n indicates the number of test observations in that fold. Higher bars indicate greater relevance of the permuted feature group for predicting daily PHQ-2 scores in that specific temporal window.

## Participant F

**Figure S9.** Eligibility analysis using repeated time-series cross-validation. Top panels show the elastic net model (orange) and constant baseline model (grey) evaluated across all repeated cross-validation folds for participant F. Left: coefficient of determination (R²) per fold. Right: mean absolute error (MAE) per fold. Bottom panels show performance improvements relative to the baseline: left, ΔR² = R²(EN) − R²(Dummy); right, ΔMAE = MAE(Dummy) − MAE(EN). Positive values indicate better performance than the baseline model. Fold-wise improvements serve as the basis for the bootstrap significance test that determines eligibility status.

**Figure S10.** Eligibility analysis using repeated time-series cross-validation. Top panels show the random forest model (red) and constant baseline model (grey) evaluated across all repeated cross-validation folds for participant F. Left: coefficient of determination (R²) per fold. Right: mean absolute error (MAE) per fold. Bottom panels show performance improvements relative to the baseline: left, ΔR² = R²(EN) − R²(Dummy); right, ΔMAE = MAE(Dummy) − MAE(EN). Positive values indicate better performance than the baseline model. Fold-wise improvements serve as the basis for the bootstrap significance test that determines eligibility status.

**Figure S11.** Fold-wise permutation importance for elastic net in participant F. Relative feature importance across rolling time-based folds for feature groups. Bars represent the proportional performance degradation (relative ΔMAE) caused by permuting each feature group within 7-day blocks in the corresponding fold. Labels on the top of the bars show the R² of the elastic net model calculated on the test observations for each fold (red if negative), and n indicates the number of test observations in that fold. Higher bars indicate greater relevance of the permuted feature group for predicting daily PHQ-2 scores in that specific temporal window.

**Figure S12.** Fold-wise permutation importance for random forest in participant F. Relative feature importance across rolling time-based folds for feature groups. Bars represent the proportional performance degradation (relative ΔMAE) caused by permuting each feature group within 7-day blocks in the corresponding fold. Labels on the top of the bars show the R² of the elastic net model calculated on the test observations for each fold (red if negative), and n indicates the number of test observations in that fold. Higher bars indicate greater relevance of the permuted feature group for predicting daily PHQ-2 scores in that specific temporal window.

## Participant G

**Figure S13.** Eligibility analysis using repeated time-series cross-validation. Top panels show the elastic net model (orange) and constant baseline model (grey) evaluated across all repeated cross-validation folds for participant G. Left: coefficient of determination (R²) per fold. Right: mean absolute error (MAE) per fold. Bottom panels show performance improvements relative to the baseline: left, ΔR² = R²(EN) − R²(Dummy); right, ΔMAE = MAE(Dummy) − MAE(EN). Positive values indicate better performance than the baseline model. Fold-wise improvements serve as the basis for the bootstrap significance test that determines eligibility status.

**Figure S14.** Eligibility analysis using repeated time-series cross-validation. Top panels show the random forest model (red) and constant baseline model (grey) evaluated across all repeated cross-validation folds for participant G. Left: coefficient of determination (R²) per fold. Right: mean absolute error (MAE) per fold. Bottom panels show performance improvements relative to the baseline: left, ΔR² = R²(EN) − R²(Dummy); right, ΔMAE = MAE(Dummy) − MAE(EN). Positive values indicate better performance than the baseline model. Fold-wise improvements serve as the basis for the bootstrap significance test that determines eligibility status.

**Figure S15.** Fold-wise permutation importance for elastic net in participant G. Relative feature importance across rolling time-based folds for feature groups. Bars represent the proportional performance degradation (relative ΔMAE) caused by permuting each feature group within 7-day blocks in the corresponding fold. Labels on the top of the bars show the R² of the elastic net model calculated on the test observations for each fold (red if negative), and n indicates the number of test observations in that fold. Higher bars indicate greater relevance of the permuted feature group for predicting daily PHQ-2 scores in that specific temporal window.

**Figure S16.** Fold-wise permutation importance for random forest in participant G. Relative feature importance across rolling time-based folds for feature groups. Bars represent the proportional performance degradation (relative ΔMAE) caused by permuting each feature group within 7-day blocks in the corresponding fold. Labels on the top of the bars show the R² of the elastic net model calculated on the test observations for each fold (red if negative), and n indicates the number of test observations in that fold. Higher bars indicate greater relevance of the permuted feature group for predicting daily PHQ-2 scores in that specific temporal window.

## Participant H

**Figure S17.** Eligibility analysis using repeated time-series cross-validation. Top panels show the elastic net model (orange) and constant baseline model (grey) evaluated across all repeated cross-validation folds for participant H. Left: coefficient of determination (R²) per fold. Right: mean absolute error (MAE) per fold. Bottom panels show performance improvements relative to the baseline: left, ΔR² = R²(EN) − R²(Dummy); right, ΔMAE = MAE(Dummy) − MAE(EN). Positive values indicate better performance than the baseline model. Fold-wise improvements serve as the basis for the bootstrap significance test that determines eligibility status.

**Figure S18.** Eligibility analysis using repeated time-series cross-validation. Top panels show the random forest model (red) and constant baseline model (grey) evaluated across all repeated cross-validation folds for participant H. Left: coefficient of determination (R²) per fold. Right: mean absolute error (MAE) per fold. Bottom panels show performance improvements relative to the baseline: left, ΔR² = R²(EN) − R²(Dummy); right, ΔMAE = MAE(Dummy) − MAE(EN). Positive values indicate better performance than the baseline model. Fold-wise improvements serve as the basis for the bootstrap significance test that determines eligibility status.

## Participant I

**Figure S19.** Eligibility analysis using repeated time-series cross-validation. Top panels show the elastic net model (orange) and constant baseline model (grey) evaluated across all repeated cross-validation folds for participant I. Left: coefficient of determination (R²) per fold. Right: mean absolute error (MAE) per fold. Bottom panels show performance improvements relative to the baseline: left, ΔR² = R²(EN) − R²(Dummy); right, ΔMAE = MAE(Dummy) − MAE(EN). Positive values indicate better performance than the baseline model. Fold-wise improvements serve as the basis for the bootstrap significance test that determines eligibility status.

**Figure S20.** Eligibility analysis using repeated time-series cross-validation. Top panels show the random forest model (red) and constant baseline model (grey) evaluated across all repeated cross-validation folds for participant I. Left: coefficient of determination (R²) per fold. Right: mean absolute error (MAE) per fold. Bottom panels show performance improvements relative to the baseline: left, ΔR² = R²(EN) − R²(Dummy); right, ΔMAE = MAE(Dummy) − MAE(EN). Positive values indicate better performance than the baseline model. Fold-wise improvements serve as the basis for the bootstrap significance test that determines eligibility status.

**Figure S21.** Fold-wise permutation importance for elastic net in participant I. Relative feature importance across rolling time-based folds for feature groups. Bars represent the proportional performance degradation (relative ΔMAE) caused by permuting each feature group within 7-day blocks in the corresponding fold. Labels on the top of the bars show the R² of the elastic net model calculated on the test observations for each fold (red if negative), and n indicates the number of test observations in that fold. Higher bars indicate greater relevance of the permuted feature group for predicting daily PHQ-2 scores in that specific temporal window.

**Figure S22.** Fold-wise permutation importance for random forest in participant I. Relative feature importance across rolling time-based folds for feature groups. Bars represent the proportional performance degradation (relative ΔMAE) caused by permuting each feature group within 7-day blocks in the corresponding fold. Labels on the top of the bars show the R² of the elastic net model calculated on the test observations for each fold (red if negative), and n indicates the number of test observations in that fold. Higher bars indicate greater relevance of the permuted feature group for predicting daily PHQ-2 scores in that specific temporal window.

## Participant J

**Figure S23.** Eligibility analysis using repeated time-series cross-validation. Top panels show the elastic net model (orange) and constant baseline model (grey) evaluated across all repeated cross-validation folds for participant J. Left: coefficient of determination (R²) per fold. Right: mean absolute error (MAE) per fold. Bottom panels show performance improvements relative to the baseline: left, ΔR² = R²(EN) − R²(Dummy); right, ΔMAE = MAE(Dummy) − MAE(EN). Positive values indicate better performance than the baseline model. Fold-wise improvements serve as the basis for the bootstrap significance test that determines eligibility status.

**Figure S24.** Eligibility analysis using repeated time-series cross-validation. Top panels show the random forest model (red) and constant baseline model (grey) evaluated across all repeated cross-validation folds for participant J. Left: coefficient of determination (R²) per fold. Right: mean absolute error (MAE) per fold. Bottom panels show performance improvements relative to the baseline: left, ΔR² = R²(EN) − R²(Dummy); right, ΔMAE = MAE(Dummy) − MAE(EN). Positive values indicate better performance than the baseline model. Fold-wise improvements serve as the basis for the bootstrap significance test that determines eligibility status.

**Figure S25.** Fold-wise permutation importance for elastic net in participant J. Relative feature importance across rolling time-based folds for feature groups. Bars represent the proportional performance degradation (relative ΔMAE) caused by permuting each feature group within 7-day blocks in the corresponding fold. Labels on the top of the bars show the R² of the elastic net model calculated on the test observations for each fold (red if negative), and n indicates the number of test observations in that fold. Higher bars indicate greater relevance of the permuted feature group for predicting daily PHQ-2 scores in that specific temporal window.

**Figure S26.** Fold-wise permutation importance for random forest in participant J. Relative feature importance across rolling time-based folds for feature groups. Bars represent the proportional performance degradation (relative ΔMAE) caused by permuting each feature group within 7-day blocks in the corresponding fold. Labels on the top of the bars show the R² of the elastic net model calculated on the test observations for each fold (red if negative), and n indicates the number of test observations in that fold. Higher bars indicate greater relevance of the permuted feature group for predicting daily PHQ-2 scores in that specific temporal window.

## Participant K

**Figure S27.** Eligibility analysis using repeated time-series cross-validation. Top panels show the elastic net model (orange) and constant baseline model (grey) evaluated across all repeated cross-validation folds for participant K. Left: coefficient of determination (R²) per fold. Right: mean absolute error (MAE) per fold. Bottom panels show performance improvements relative to the baseline: left, ΔR² = R²(EN) − R²(Dummy); right, ΔMAE = MAE(Dummy) − MAE(EN). Positive values indicate better performance than the baseline model. Fold-wise improvements serve as the basis for the bootstrap significance test that determines eligibility status.

**Figure S28.** Eligibility analysis using repeated time-series cross-validation. Top panels show the random forest model (red) and constant baseline model (grey) evaluated across all repeated cross-validation folds for participant K. Left: coefficient of determination (R²) per fold. Right: mean absolute error (MAE) per fold. Bottom panels show performance improvements relative to the baseline: left, ΔR² = R²(EN) − R²(Dummy); right, ΔMAE = MAE(Dummy) − MAE(EN). Positive values indicate better performance than the baseline model. Fold-wise improvements serve as the basis for the bootstrap significance test that determines eligibility status.

**Figure S29.** Fold-wise permutation importance for elastic net in participant K. Relative feature importance across rolling time-based folds for feature groups. Bars represent the proportional performance degradation (relative ΔMAE) caused by permuting each feature group within 7-day blocks in the corresponding fold. Labels on the top of the bars show the R² of the elastic net model calculated on the test observations for each fold (red if negative), and n indicates the number of test observations in that fold. Higher bars indicate greater relevance of the permuted feature group for predicting daily PHQ-2 scores in that specific temporal window.

**Figure S30.** Fold-wise permutation importance for random forest in participant K. Relative feature importance across rolling time-based folds for feature groups. Bars represent the proportional performance degradation (relative ΔMAE) caused by permuting each feature group within 7-day blocks in the corresponding fold. Labels on the top of the bars show the R² of the elastic net model calculated on the test observations for each fold (red if negative), and n indicates the number of test observations in that fold. Higher bars indicate greater relevance of the permuted feature group for predicting daily PHQ-2 scores in that specific temporal window.

## Participant M

**Figure S31.** Eligibility analysis using repeated time-series cross-validation. Top panels show the elastic net model (orange) and constant baseline model (grey) evaluated across all repeated cross-validation folds for participant M. Left: coefficient of determination (R²) per fold. Right: mean absolute error (MAE) per fold. Bottom panels show performance improvements relative to the baseline: left, ΔR² = R²(EN) − R²(Dummy); right, ΔMAE = MAE(Dummy) − MAE(EN). Positive values indicate better performance than the baseline model. Fold-wise improvements serve as the basis for the bootstrap significance test that determines eligibility status.

**Figure S32.** Eligibility analysis using repeated time-series cross-validation. Top panels show the random forest model (red) and constant baseline model (grey) evaluated across all repeated cross-validation folds for participant M. Left: coefficient of determination (R²) per fold. Right: mean absolute error (MAE) per fold. Bottom panels show performance improvements relative to the baseline: left, ΔR² = R²(EN) − R²(Dummy); right, ΔMAE = MAE(Dummy) − MAE(EN). Positive values indicate better performance than the baseline model. Fold-wise improvements serve as the basis for the bootstrap significance test that determines eligibility status.

**Figure S33.** Fold-wise permutation importance for elastic net in participant M. Relative feature importance across rolling time-based folds for feature groups. Bars represent the proportional performance degradation (relative ΔMAE) caused by permuting each feature group within 7-day blocks in the corresponding fold. Labels on the top of the bars show the R² of the elastic net model calculated on the test observations for each fold (red if negative), and n indicates the number of test observations in that fold. Higher bars indicate greater relevance of the permuted feature group for predicting daily PHQ-2 scores in that specific temporal window.

**Figure S34.** Fold-wise permutation importance for random forest in participant M. Relative feature importance across rolling time-based folds for feature groups. Bars represent the proportional performance degradation (relative ΔMAE) caused by permuting each feature group within 7-day blocks in the corresponding fold. Labels on the top of the bars show the R² of the elastic net model calculated on the test observations for each fold (red if negative), and n indicates the number of test observations in that fold. Higher bars indicate greater relevance of the permuted feature group for predicting daily PHQ-2 scores in that specific temporal window.

## Participant N

**Figure S35.** Eligibility analysis using repeated time-series cross-validation. Top panels show the elastic net model (orange) and constant baseline model (grey) evaluated across all repeated cross-validation folds for participant N. Left: coefficient of determination (R²) per fold. Right: mean absolute error (MAE) per fold. Bottom panels show performance improvements relative to the baseline: left, ΔR² = R²(EN) − R²(Dummy); right, ΔMAE = MAE(Dummy) − MAE(EN). Positive values indicate better performance than the baseline model. Fold-wise improvements serve as the basis for the bootstrap significance test that determines eligibility status.

**Figure S36.** Eligibility analysis using repeated time-series cross-validation. Top panels show the random forest model (red) and constant baseline model (grey) evaluated across all repeated cross-validation folds for participant N. Left: coefficient of determination (R²) per fold. Right: mean absolute error (MAE) per fold. Bottom panels show performance improvements relative to the baseline: left, ΔR² = R²(EN) − R²(Dummy); right, ΔMAE = MAE(Dummy) − MAE(EN). Positive values indicate better performance than the baseline model. Fold-wise improvements serve as the basis for the bootstrap significance test that determines eligibility status.

## Participant O

**Figure S37.** Eligibility analysis using repeated time-series cross-validation. Top panels show the elastic net model (orange) and constant baseline model (grey) evaluated across all repeated cross-validation folds for participant O. Left: coefficient of determination (R²) per fold. Right: mean absolute error (MAE) per fold. Bottom panels show performance improvements relative to the baseline: left, ΔR² = R²(EN) − R²(Dummy); right, ΔMAE = MAE(Dummy) − MAE(EN). Positive values indicate better performance than the baseline model. Fold-wise improvements serve as the basis for the bootstrap significance test that determines eligibility status.

**Figure S38.** Eligibility analysis using repeated time-series cross-validation. Top panels show the random forest model (red) and constant baseline model (grey) evaluated across all repeated cross-validation folds for participant O. Left: coefficient of determination (R²) per fold. Right: mean absolute error (MAE) per fold. Bottom panels show performance improvements relative to the baseline: left, ΔR² = R²(EN) − R²(Dummy); right, ΔMAE = MAE(Dummy) − MAE(EN). Positive values indicate better performance than the baseline model. Fold-wise improvements serve as the basis for the bootstrap significance test that determines eligibility status.

**Figure S39.** Fold-wise permutation importance for elastic net in participant O. Relative feature importance across rolling time-based folds for feature groups. Bars represent the proportional performance degradation (relative ΔMAE) caused by permuting each feature group within 7-day blocks in the corresponding fold. Labels on the top of the bars show the R² of the elastic net model calculated on the test observations for each fold (red if negative), and n indicates the number of test observations in that fold. Higher bars indicate greater relevance of the permuted feature group for predicting daily PHQ-2 scores in that specific temporal window.

**Figure S40.** Fold-wise permutation importance for random forest in participant O. Relative feature importance across rolling time-based folds for feature groups. Bars represent the proportional performance degradation (relative ΔMAE) caused by permuting each feature group within 7-day blocks in the corresponding fold. Labels on the top of the bars show the R² of the elastic net model calculated on the test observations for each fold (red if negative), and n indicates the number of test observations in that fold. Higher bars indicate greater relevance of the permuted feature group for predicting daily PHQ-2 scores in that specific temporal window.
