# Supplementary material for: Individual-Level Modeling of Depressive Symptom Severity Using Smartphone and Wearable Data: Time-Aware 1-Year Study With Feature-Group Contributions
Source: JMIR Form Res. 2026 Aug 25;10:e99204. doi: 10.2196/99204 (PMC13507524; doi:10.2196/99204)
Supplement: Supplementary file 5 [file formative-v10-e99204-s005.docx]

*Sensitivity analysis excluding participant I. Comparison of aggregated feature-group importance profiles and Wilcoxon signed-rank test results for elastic net and random forest models with and without participant I.*
